# Gender Congruency Effects in Spanish: Behavioral Evidence from Noun Phrase Production

**DOI:** 10.3390/brainsci13040696

**Published:** 2023-04-21

**Authors:** Ruixue Wu, Niels O. Schiller

**Affiliations:** 1 Leiden University Centre for Linguistics (LUCL), Leiden University, Reuvensplaats 3, 2311 BE Leiden, The Netherlands; 2 Leiden Institute for Brain and Cognition (LIBC), Leiden University, LUMC, Postzone C2-S, P.O. Box 9600, 2300 RC Leiden, The Netherlands

**Keywords:** language production, lexico-syntactic features, gender congruency effect, PWI, Spanish

## Abstract

Grammatical gender as a lexico-syntactic feature has been well explored, and the gender congruency effect has been observed in many languages (e.g., Dutch, German, Croatian, Czech, etc.). Yet, so far, this effect has not been found in Romance languages such as Italian, French, and Spanish. It has been argued that the absence of the effect in Romance languages is due the fact that the gender-marking definite article is not exclusively dependent on the grammatical gender of the head noun, but also on its onset phonology (e.g., *lo zucchero* is ‘the sugar’ in Italian, not *il zucchero*, il being the default masculine determiner in Italian). For Spanish, this argument has also been made because feminine words starting with a stressed /a/ take the masculine article (e.g., *el água* is ‘the water’, not *la água*). However, the number of words belonging to that set is rather small in Spanish, and it may be questionable whether or not this feature can be taken as an argument for the absence of a gender congruency effect in Spanish. In this study, we investigated the gender congruency effect in native Spanish noun phrase production. We measured 30 native Spanish speakers’ naming latencies in four conditions via the picture–word interference paradigm by manipulating gender congruency (i.e., gender-congruent vs. gender-incongruent) and semantic relatedness (i.e., semantically related vs. semantically unrelated). The results revealed significantly longer naming latencies in gender-incongruent and semantically related conditions compared to gender-congruent and semantically unrelated conditions. This result suggests that grammatical gender as a lexico-syntactic feature in Spanish is used to competitively select determiners in native Spanish speakers’ noun phrases. Our findings provide an important behavioral piece of evidence for the gender congruency effect in Romance languages.

## 1. Introduction

The study of grammatical gender processing has been a topic of interest for psycholinguists for decades. In gendered languages, such as Romance languages (e.g., Spanish, Italian, etc.) and Germanic languages (e.g., German, Dutch, etc.), grammatical gender processing is a crucial part of successfully producing a determiner noun phrase (NP) (e.g., in Spanish, ‘la manzana’ (the_fem_ apple_fem_)). According to Levelt et al.’s speech production model [1], speakers need to encode the to-be-produced word by conceptualizing the message first. Then, by lexicalizing the concept, the word’s grammatical properties such as its syntactic features (e.g., grammatical gender, number, case, etc.) are activated and eventually retrieved, and its corresponding phonological and phonetic form are encoded. Finally, the phonetic motor representation of the word is articulated.

In a picture–word interference task, speakers are presented with a target picture and a distractor word. When speakers produce a determiner–NP in gender-marking languages, the concept and form of the target picture and the distractor, respectively, are activated first. Then, the syntactic representation of the target and distractor (via its word form), including the syntactic features (e.g., grammatical gender, number, case, etc.), are activated. In this case, all nodes that are conceptually related to the target are activated to different degrees, with the lexical node that is conceptually related to the target being the most highly activated. The most highly activated lexical node is then selected for production, including the retrieval of the phonological features for further word-form encoding. Finally, the determiner–NP is produced. According to this model, the gender feature of the to-be-produced word will be activated but only selected if it is needed for further production (e.g., for determiner–NP production, but not for bare noun naming, when the gender feature is not strictly needed for production).

The processing of grammatical gender in this speech production model is supported by numerous experimental studies of NP production in gendered languages such as German [2,3,4,5,6,7] and Dutch [5,6,8,9,10] (for a detailed description, see the next section). However, conflicts were observed in experimental studies in Romance languages (e.g., Spanish [11], Italian [12,13,14], Catalan [11], French [15], and Portuguese [16]), in which the correct grammatical gender was selected and produced in NP production but no effect of this selection process was found. Therefore, the question arises as to why this selection/competition of grammatical gender is not reflected by a gender congruency effect in Romance languages. This study tackles this question experimentally.

### 1.1. The Gender Congruency Effect

Gender agreement, generally represented by agreement between the noun and the determiner or adjective in the noun phrase [17], is a key feature of gender-marking languages such as Romance languages (e.g., Spanish) as well as Germanic languages (e.g., German and Dutch). Nouns in these languages are assigned a gender (e.g., in Spanish, masculine or feminine), which is marked on associated determiners and adjectives, for example, in Spanish, ‘la manzana roja’, (literally: the_fem_ apple_fem_ red_fem_). In this example, the form of the determiner is ‘la’ when ‘manzana’ is a feminine noun. In other words, the determiners match the gender of the noun they accompany. The gender congruency effect, which entails faster and more accurate processing in cases of a match between the gender of nouns and their associated determiners or adjectives, has been studied extensively in Romance languages [16,18,19,20], as well as in German [2,3,4,5,6,7], Dutch [5,6,8,9,10] and some other gendered languages (for an overview, see Wang and Schiller [21] and Sá-Leite et al. [22]; for a recent meta-analysis, see Bürki et al., in press [23]).

The gender congruency effect in language production has been investigated in experimental studies using the picture–word interference (PWI) paradigm [24,25,26]. In this experimental paradigm, participants are asked to name a picture while ignoring a distractor word presented shortly before, at the same time, or shortly after picture onset. It has been found that the reaction time to name the picture is affected by the relationship between the distractor and the target picture. In the study of Schriefers [26], the PWI task was initially employed to investigate how grammatical gender (i.e., in Dutch, common and neuter) is processed by native Dutch speakers. He manipulated the gender congruency between target pictures and distractors, i.e., creating gender-congruent conditions (e.g., a target picture of a ‘boek,’ book_neuter_, with the distractor ‘dak,’ roof_neuter_) and gender-incongruent conditions (e.g., a target picture of a ‘boek,’ book_neuter_, with the distractor ‘tafel,’ table_common_). Participants were presented with a target picture along with a gender-congruent or -incongruent distractor at the same time and asked to name the picture using a noun phrase while ignoring the distractor. Faster naming latencies were obtained in the gender-congruent condition than in the gender-incongruent condition, coined as the gender congruency effect. Schriefers [26] interpreted the gender congruency effect as the result of grammatical gender features of targets and distractors competing for selection in participants’ noun phrase production in gender-incongruent conditions.

Experimental research has shown a consistently faster response time for gender-congruent conditions than for gender-incongruent conditions in noun phrase production in German [2,3,4,5,6,7] and Dutch [5,6,8,9,10]. Bürki et al. [7], for instance, conducted a picture naming task in German using the PWI paradigm by manipulating two factors, i.e., gender congruency and phonological congruency. Participants were asked to name the pictures using noun phrases and ignore the distractors. As the grammatical gender of the target picture is selected in competition with distractors during NP production (determiner + noun or determiner + adjective + noun), variations in the naming response times were found to depend on the gender and phonological congruency status. Both the gender-congruent condition and phonologically congruent condition were faster than the corresponding incongruent conditions. The consistent gender congruency effect was found in many studies in the NP language production of German [2,3,4,5,6,7] and Dutch [5,6,8,9,10] (for an overview, see Wang and Schiller [21] and Sá-Leite et al. [22]).

Nevertheless, conflicts have been observed in the attempts to replicate the gender congruency effect in Romance languages. The gender congruency effect in Italian was successfully replicated in the production of bare nouns (e.g., in Paolieri et al. [18,27] and Cubelli et al. [19]), but not in the production of noun phrases (e.g., ‘il gatto’ (the cat)) in Cubelli et al.’s research [19]. In Cubelli et al.’s study [19], a gender congruency effect with an unexpected direction was found in Italian bare noun production. Longer naming latencies were observed in the gender-congruent condition than in the gender-incongruent condition. This effect has been successfully replicated in three experiments with different materials (e.g., in Paolieri et al. [18,27]). However, Finocchiaro et al. [28] reported the absence of a gender congruency effect in their experimental work on Italian, Spanish, and French using bare noun naming. They attempted to replicate the study of Cubelli et al. [19] by testing native Italian speakers on bare noun production. However, no gender congruency effect was found with either transparent or opaque distractors in two experiments. Similarly, naming latencies in their Spanish and French bare noun production experiments were not affected by the gender of a distractor word presented with the target picture. On the contrary, Alario and Caramazza [15] demonstrated significantly faster response times for gender-congruent conditions than for incongruent conditions in French NP production (e.g., determiner + noun and determiner + adjective + noun).

Moreover, O’Rourke’s [29] and Finocchiaro et al.’s [28] replication studies did not result in a finding of gender congruency effects in Spanish bare noun production, but Paolieri et al. [27] did. Furthermore, Paolieri et al. [27] found a reversed gender congruency effect, i.e., participants responded faster when naming target pictures in Italian (e.g., ‘pera’ (pear_fem_)) with gender-incongruent distractors (e.g., ‘cervo’ (deer_mas_)) than with gender-congruent distractors (e.g., ‘calza’ (sock_fem_)). Similarly, they also observed longer naming latencies in Spanish for target pictures (e.g., ‘mono’ (monkey_mas_)) with gender-matched distractors (e.g., ‘grifo’ (tap_mas_)) than with gender-unmatched distractors (e.g., ‘cartera’ (wallet_fem_)). Additionally, Von Grebmer zu Wolfsthurn et al. [20] observed a cross-language gender congruency effect in Spanish NP production with German speakers who were learning Spanish as a second language. Based on these contradictory findings in Romance languages, it is at least questionable whether or not the selection process of grammatical gender is competitive, and if so, whether or not this competitive process surfaces as a variation in naming latencies. In particular, the answer to the question of whether or not the congruency status between the grammatical gender of the targets and distractors has a significant effect on naming latencies remains unclear.

### 1.2. The Grammatical Gender Feature in Spanish

Spanish, like many other Romance languages, has a gender system that distinguishes between masculine and feminine genders for nouns and their associated determiners and adjectives. Specifically, Spanish has a two-gender system including two-gender features for nouns (masculine and feminine), with the determiners and adjectives exhibiting gender agreement according to the lexical properties of the following nouns in NPs (e.g., determiner + noun and determiner + adjective + noun) [29,30]. The distribution of feminine and masculine gender values is approximately balanced in Spanish [31,32]. However, it has been argued that masculine and feminine gender may be represented differentially in Spanish, with masculine being the default gender, and feminine taking a more marked position in the language [33].

As a lexico-syntactic feature, grammatical gender in Spanish applies to all nouns, and the grammatical gender agreement of determiners is obligatory within NPs (e.g., ‘la camisa’ (the_fem_ shirt_fem_)) [21]. Many nouns are morphologically and/or phonologically marked by grammatical gender [21], and the selection of determiners and adjectives depends on the phonological and morphological forms of nouns [12,34]. Specifically, nouns ending in ‘-o’ often have masculine gender (99.9%) and those ending in ‘-a’ generally have feminine gender (96.3%). Additionally, a small number of nouns, i.e., those ending in ‘-e’, have feminine or masculine gender, e.g., ‘el tigre’ (the_mas_ tiger_mas_) and ‘la llave’ (the_fem_ key_fem_), with 89.4% of all ‘-e’ words being masculine [29,35,36]. Similarly, a small number of nouns ending in consonants (e.g., ‘-z’, ‘-l’, ‘-s’, etc.) are opaque [29]. In general, there are about twice as many transparent nouns as there are opaque nouns in Spanish [37].

According to these transparent endings, the selection of determiners can mainly rely on the morphological feature of nouns, in which the corresponding feminine determiners (e.g., ‘la’ (the_singular_) and ‘las’ (the_plural_)) are assigned to nouns ending in ‘-a’ (e.g., ‘la guitarra’ (the_fem_ guitar_fem_)). Similarly, the masculine determiners (e.g., ‘el’ (the_singular_) and ‘los’ (the_plural_)) are involved in nouns ending in ‘-o’ (e.g., ‘el gato’ (the_mas_ cat_mas_)). However, there are less than 0.5% exceptions to this transparent gender marking of nouns, including words where the correspondence between their gender and their ending is not transparent [11]. For instance, the feminine determiners cannot be assigned to nouns beginning with a stressed /a/ (e.g., ‘el água’ (the_mas_ water_mas_)) [11]. This means that only when the phonological information about the nouns is available can the correct form of the determiner be selected. Whether or not the contradictory findings of the gender congruency effect in Spanish are due to the fact that the gender-marking determiners are not exclusively dependent on the grammatical gender of the head noun, but also on its onset phonology, invites more debates.

### 1.3. The Current Study: Native Noun Phrase Production in Spanish

This study presents behavioral evidence from a determiner–noun phrase production task using a picture–word interference (PWI) paradigm [24,25] to further explore the gender congruency effect in Spanish. We set gender congruency (i.e., gender-congruent vs. gender-incongruent) and semantic relatedness (i.e., semantically related vs. semantically unrelated) as the two main factors. The goal of the present study is to investigate whether or not the grammatical gender of determiners is competitively selected in the production of noun phrases by native Spanish speakers in a well-controlled experiment with new targets and pictures. If grammatical gender is selected competitively, the next question is whether or not this competition is reflected in the effect of reaction times. Therefore, we addressed the main research question: is there a gender congruency effect in native Spanish NP production?

#### Hypotheses

The lexical selection by competition theory [1,38] refers to the process of competitively selecting the target word from all the activated non-target words when producing a word. Speakers will take more time to select the target word in their language production when more non-target words are highly activated. Based on this theory, we expect the effects of semantic relatedness on naming latencies to be present in the picture naming task. That is, a slower reaction time is expected when the target picture and the distractor belong to the same semantic category [39,40]. On the contrary, we predict faster naming latencies in semantically unrelated conditions, in which the target object belongs to a different semantic category than the distractor. For the gender congruency condition, we predict a significant gender congruency effect on naming latencies. Specifically, we predict that the gender-congruent condition shortens speakers’ reaction times for naming target objects. In contrast, speakers’ naming latencies are prolonged due to the gender difference between the target and the distractor.

Levelt et al.’s model of speech production [1] claims that the content of the target picture is conceptualized first at the conceptual level, and then the grammatical gender as a syntactic feature is activated and selected at the lemma level, both occurring in a sequential order in the picture naming task. Since the selection of the grammatical gender of determiners in NPs is dominated by properties of the noun in Spanish [11], the process of grammatical gender is supposed to be independent of semantic relatedness in word production. As a result, an interaction between the gender congruency effect and the semantic interference is not expected.

## 2. Materials and Methods

### 2.1. Participants

Thirty healthy, right-handed native Spanish speakers (M_age_ = 26.08 years and SD_age_ = 4.85 years; nineteen females) participated in this experiment. Participants did not report any history of neurological or language disorders. Before testing, informed consent was obtained from all participants. They read an information sheet and signed a consent form, which was approved by the Ethics committee of the Faculty of Humanities at Leiden University. Upon termination of all tasks, participants were paid for their participation.

### 2.2. Materials

Twenty black-and-white line pictures were obtained from Severens’ picture database [41] based on two criteria: pictures had to refer to a familiar and concrete concept and pictures had to have easily recognizable features. Each picture was assigned four distractors, which were manipulated in four conditions according to their gender congruency (i.e., gender-congruent vs. gender-incongruent) with and semantic relatedness (i.e., semantically related vs. semantically unrelated) to the target picture (see Appendix A). As a result, a total of 80 combinations of target picture and distractor pairs were generated. The word frequency of the distractors was controlled on the basis of *Corpus del Español* [42] across the four conditions, with F(3, 76) = 1.358 and *p* = 0.262. Similarly, the visual complexity of the distractors was controlled by balancing the number of letters across the four conditions, with F(3, 76) = 1.925 and *p* = 0.133. Targets and distractors were neither phonologically nor orthographically related.

### 2.3. Design and Procedure

The experiment was designed as a 2 by 2 fully factorial within-subjects design with two main factors: gender congruency (G) and semantic relatedness (S). The factor gender congruency included two levels, i.e., gender-congruent (G+) and gender-incongruent (G−), based on gender congruency or gender incongruency between the target picture and the distractors. The factor semantic relatedness was divided into two levels, i.e., semantically related (S+) or semantically unrelated (S−), depending on whether or not the target pictures and the distractors belonged to the same semantic category. As a result, four conditions were generated for each target picture: G+S+, G+S−, G−S+, and G−S− (see Table 1).

The picture naming task was programmed in E-prime 2.0 [43] and designed based on the picture–word interference (PWI) paradigm. In order to counterbalance the effect of order, we used the Windows program Mix [44] to generate a pseudo-random order of trials according to two criteria: no two trials within the same condition or associated with the same target picture were allowed to appear consecutively, and trials of the same grammatical gender could be presented no more than twice in a row. As a result, 40 blocks were generated in E-prime 2.0. Furthermore, we also used the by-subjects order design for the programming of the task, so that the order of the blocks was randomized across participants.

**Figure 1:**
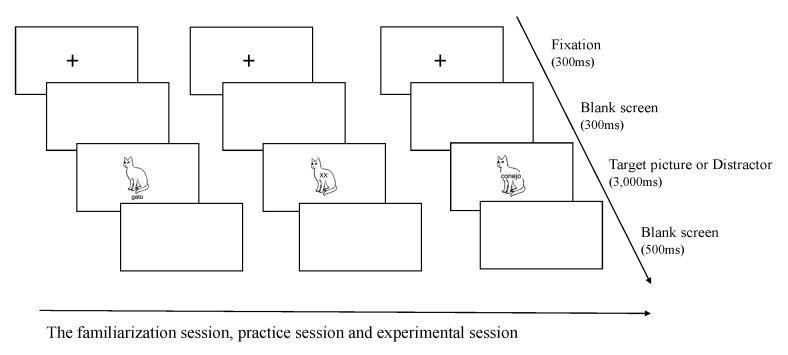
Illustration of the familiarization session, practice session and experimental session for the picture naming task.

The whole experiment was divided into three sessions: a familiarization session, a practice session, and an experimental session, lasting 20 min in total. Participants were first presented with the familiarization session, in which they were instructed to learn the exact name under the target picture for 3000 ms. After the presentation of all 20 target pictures, participants were asked to practice naming the same pictures in a practice session, in which the target pictures were presented with a meaningless ‘XX’ string in the center of the screen. In this session, each picture was presented for 3000 ms. The correct name of the target was provided if participants produced an incorrect name. In the experimental session, participants were expected to name the target picture as fast and accurately as possible with a Spanish noun phrase (e.g., ‘el gato’ (the_mas_ cat_mas_)) while ignoring the distractor word. For each trial, the typical procedure began with a fixation cross presented in the center of the screen for 300 ms, followed by a blank screen for 300 ms. This was followed by the display of the target picture and distractor word for 3000 ms, during which participants’ vocal responses were recorded. At the end of each trial, a blank screen was displayed for 500 ms (see Figure 1).

## 3. Results

Naming latencies were calculated and extracted from all recorded the data of 30 participants using Praat [45] (see Table 2). Of all the 2400 recorded data points, 4.6% was removed from the further behavioral data analysis due to the presence of (a) incorrect responses, no responses, or delayed responses (3.3%) and (b) outliers, i.e., naming latencies exceeding 3 SDs around the average responding time of participants (1.3%). Next, we employed the generalized linear mixed model (GLMM) and the glmer function with a gamma distribution to analyze our behavioral data in Rstudio version 4.2.2 (Vienna, Austria) [46] using the lme4 package [47].

**Figure 2:**
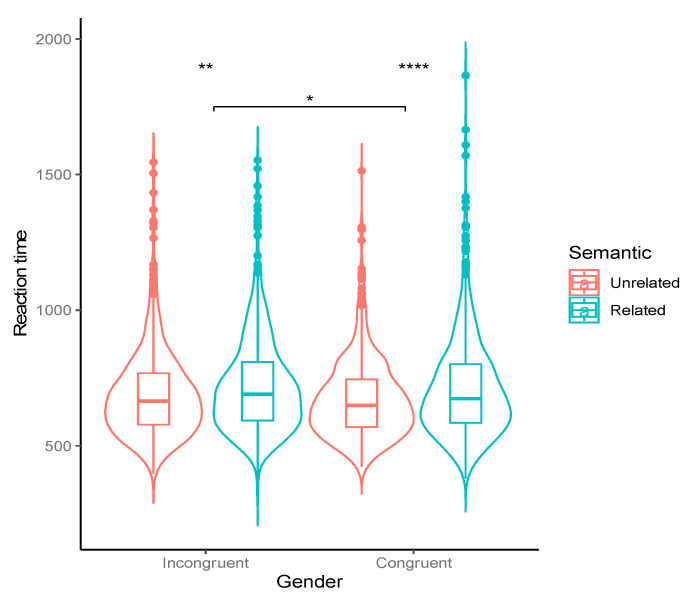
Picture naming latencies by condition. Both gender congruency and semantic relatedness showed a significant effect on naming latencies (i.e., *p* < 0.0001, indicated as “****”; *p* < 0.01, indicated as “**” and *p* < 0.05, indicated as “*” respectively in the figure).

To avoid the risk of increasing the Type I error rate, the analysis of the behavioral data was modeled using a top–down model selection procedure [48], in which the model starts with the theoretically maximal model. In order to fit the model to our data, we included gender congruency and semantic relatedness as two fixed factors, and item and participant as two random factors. To control for potential confounders, we added a distractor category as a co-variate to our statistical analysis. Further, we included a target picture category as a random slope for the item factor. We generated the best-fit model for our data by taking the following steps: first, we removed the non-significant random effect of interaction between gender congruency and semantic relatedness for each participant, and the interaction of the fixed effects of gender congruency and semantic relatedness in the case of a singular fit; second, the random slopes of the target category for the items, and the distractor category were removed as they did not significantly improve the model fit and resulted in non-convergence; third, the correlation between gender congruency and semantic relatedness for the participant factor, and the random intercept and slope of gender congruency for the participant factor were excluded on the basis of akaike’s information criterion (AIC) [49], the Bayesian information criterion (BIC) [50], and the log-likelihood ratio; finally, the best-fit model for our data was fitted with gender congruency and semantic relatedness as fixed effects, and random intercepts for the participant and target item, as well as a by-participant random slope for semantic relatedness. The best-fit model (see Table 3) demonstrated significantly shorter naming latencies in gender-congruent conditions compared to gender-incongruent conditions with β = −13.526, SE = 4.64, t = −2.91, and *p* = 0.004. Moreover, participants responded significantly slower to semantically related trials than to semantically unrelated trials, with β = 38.41, SE = 10.75, t = 3.57, and *p* < 0.001 (see Figure 2).

## 4. Discussion

In this study, we investigated the potential impact of gender congruency and semantic relatedness on Spanish NP production using the picture–word interference paradigm. The gender congruency and semantic relatedness were manipulated between the target picture and the distractors. The gender congruency effect was examined by comparing the naming latencies between gender-matched and gender-non-matched determiner–NPs. We predicted shorter naming latencies for gender-congruent NPs compared to incongruent NPs. Critically, this would indicate that the target gender value was activated and selected at the *lemma level*. We also studied the semantic effect with semantically related or unrelated NPs, and we expected longer response times for unrelated trials compared to related trials. The presence of such a semantic interference effect would imply that the concept of the target picture and the distractor actively competed for selection during *lemma level* processing.

In line with our expectations, we found that participants’ naming latencies were significantly affected by the semantic relatedness of a distractor word and the target picture. To be more precise, the participants showed longer naming latencies when naming a target picture with a distractor of the same semantic category. In other words, the semantic interference effect [25,39,51] was found in naming semantically related targets and distractors. Our behavioral results are important because the presence of the semantic interference effect is suggestive of the lexical selection process during semantic processing. It implies that the semantically related distractors represent competitors of the target, resulting in a more difficult selection process than in unrelated conditions. When the target is presented with a semantically related distractor, more than one lemma of the target and the distractor is activated, and some of these activated lemmas overlap with the same semantic features, leading to more competition during the lexical selection process. These behavioral findings are in line with numerous previous studies that report semantic interference [1,38,51,52].

Meanwhile, we explored the effect of gender congruency on the access to the target word in the presence of a distractor by manipulating the gender congruency between the target and the distractors. A significant gender congruency effect was observed in the naming latencies of the NP naming task. Our behavioral results suggest that participants were significantly faster at naming the target with gender-congruent distractors than with gender-incongruent distractors. These findings are important for three reasons: First, the significantly faster naming latencies in the gender-congruent conditions suggest the presence of a gender congruency effect during gender processing. Second, the gender congruency effect implies that the competition occurred during the process of gender feature selection, in which the different gender values for the target picture and the distractor were activated in the gender-incongruent condition and the gender feature of distractors competed for selection at the lemma level and interfered with the naming of the target NP. Third, the statistically significant longer reaction time in gender-incongruent conditions also suggest that the competition can be reflected by a reaction time effect in native Spanish NP production.

It is important to mention that no significant gender congruency effect was found in previous studies on Spanish NP naming (e.g., in Costa et al. [11]) and some of the bare noun naming studies (e.g., O’Rourke [29] and Finocchiaro et al. [28]). However, the results of the gender congruency effect are reversed for the study of Paolieri et al. [27], where longer naming latencies were found for gender-matched target–distractor pairs than for gender-non-matched pairs. Compared to all aspects of the previous studies, the gender congruency effect was possibly found in the current study for the following reasons.

First of all, in the study by Paolieri et al. [27], twenty native Spanish participants were asked to name 28 target pictures using bare nouns in Spanish, and a reliable reversed gender congruency effect was obtained in their bare noun naming tasks. They concluded that their findings confirm the assumption that grammatical gender is not only a syntactic feature, but also an inherent lexical property of nouns that can be selected in bare noun production. As is consistent with this assumption, in our experiment, participants were asked to perform a noun phrase naming task, in which a determiner–noun phrase had to be produced. In determiner–noun phrase production, the correct determiner–noun phrase can only be produced by selecting the grammatical gender of nouns and determiners. Therefore, it was essential for participants to explicitly produce the correct determiner when given the target gender value. In this process, there was competition in the selection of the syntactic feature of the target.

However, the gender congruency effect in the study by Paolieri et al. [27] was reversed, with the longer naming latencies being found in the gender-congruent condition. This finding is unexpected within the current language production models (e.g., the LRM model [1] and WEAVER++ model [53]). More specifically, according to the LRM model [1], when the target picture and a distractor are presented to participants, grammatical gender as an inherent lexical property of nouns is activated. In a gender-incongruent condition, different gender nodes are activated for the distractor and the target, and the gender feature of the distractor interferes with the naming of the target. In this case, the competition occurs when the target syntactic feature is selected with the interference from the distractor. In contrast, in a gender-congruent condition, the same gender node is activated for the distractor and the target, where no competition occurs. As a result, it takes more time to produce an NP with the correct determiner in the gender-incongruent condition than in the gender-congruent condition, as the target gender node competes for selection with non-target activated gender nodes in the gender-incongruent condition. In this case, the effect on naming latencies of gender congruency is easy to observe. Our results are consistent with the results of gender processing in current models.

Second, it is necessary to pay attention to the study by Costa et al. [11], who conducted three experiments asking participants to perform three Spanish NP (i.e., determiner + noun) naming tasks and found no gender congruency effect. They concluded that the gender congruency effects may have occurred during early lexical selection processes but were rendered invisible by the selection and retrieval of the phonological form of a word. However, this explanation is rather unlikely for Spanish, since only a rather small number of nouns, i.e., less than 50 cases of all lexical items (less than 0.5%) [54], are consistent with this conclusion. In general, noun endings are not only either transparent (i.e., ‘-o’ for masculine and ‘-a’ for feminine) or opaque (i.e., ‘-e’ for either masculine or feminine, or consonants, e.g., ‘-z’, ‘-l’, ‘-s’, etc.) [29]. In many cases, the correct choice of determiner in Spanish can be made on the basis of the transparent property (i.e., the phonological information) of the nouns. Only in a small proportion of cases (less than 0.5%) of all lexical items does the selection of determiners depend on the phonological context [11]. Given these facts, the conclusion of Costa et al. [11] that there is no gender congruency effect in Spanish NP production becomes less likely. On the other hand, the conclusion of Costa et al. [11] also can be interpreted in the following way: it is possible for the gender congruency effect to be found without selecting and retrieving the phonological form of a word. In our list of stimuli, there were no exceptions included (i.e., masculine determiner for nouns starting with a stressed /a/) for both targets and distractors, which meant that the selection of determiners could be based on the lexical information of the nouns. In this case, the finding of a gender congruency effect in our study seems plausible.

Third, compared to the study by O’Rourke [29], in which the distractors were presented in auditory form, we adopted a different experimental procedure with the presentation of the distractor being in printed form. Such a difference in display form has been shown to influence the timing of distractor effects [28,55]. Given this difference, the discrepancy between O’Rourke’s study [29] and the present study does not necessarily imply contradictory conclusions. Additionally, in terms of the structure of the grammatical gender of nouns, Spanish is similar to Dutch in that phonological information is hardly necessary to choose the correct determiners [11]. The few exceptional cases may be hard wired, i.e., learned and stored as phrases, e.g., *el água*, “the water”, or *el água* fria, “the cold water”. In this case, the findings of the study by O’Rourke [29] and Finocchiaro et al. [28] may be explained by the Dutch case, where the gender congruency effect was not found in bare noun production but was found in noun phrase production [8]. This process of producing NPs can be interpreted as an amplification of the potential gender congruency effect that is not detected in the simpler bare noun task, which does not require the explicit selection of the grammatical gender for determiner selection.

Additionally, it is worth mentioning the statistical analysis we applied to our data, which supports the robustness of the effects we observed in the present study. First, unlike the previous studies (e.g., the study by Cubelli et al. [19]), which defined outliers as a naming latency exceeding two standard deviations below or above the average responding time of participants (i.e., mean +/− 2 SDs), we defined outliers by removing the naming latencies that laid three standard deviations around participants’ mean response times (i.e., mean +/− 3 SDs). This resulted in few data points being identified as outliers and excluded from further data analysis. In general, the method of using mean +/− 2 SDs is considered a restrictive method that excludes outliers, resulting in the loss of more reliable data [28,56]. Second, the implementation of a single-trial generalized linear mixed-effect model (GLMM) was a novelty of our statistical analysis. Compared to the traditional statistical analysis of naming latencies using ANOVAs (e.g., that performed by Paolieri et al. [27], Finocchiaro et al. [28], etc.), the single-trial GLMM is more sensitive and specific to single-trial analysis, and does not assume an underlying distribution or an equal number of observations for each participant or condition. Instead, it captures variance as explained by each participant and by each item as well as the experimental manipulations of interest [57]. When modeling behavioral data with unequal numbers of conditions or participants, it therefore has superior explanatory power to traditional ANOVAs. In other words, it also provided a more robust result for our behavioral data analysis.

## 5. Conclusions

In this study, we explored whether or not grammatical gender, as a lexico-syntactic feature in Spanish, is used to competitively select determiners in the production of noun phrases by native Spanish speakers in a well-controlled experiment. We employed the picture–word interference paradigm to examine the naming latencies of 30 participants for multiple objects in four conditions in which gender congruency, i.e., gender-congruent and gender-incongruent, and semantic relatedness, i.e., semantically related and semantically unrelated, were manipulated. We found reliable effects of grammatical gender congruency and semantic relatedness in noun phrase production in Spanish. More specifically, statistically significant shorter naming latencies were found for gender-congruent target–distractor pairs than for gender-incongruent pairs. Similarly, we found longer naming latencies in semantically related trials than in unrelated trials. These results provide crucial evidence supporting the notion that grammatical gender in determiner–NPs is competitively selected and that this competition is reflected in speakers’ naming latencies. Furthermore, our findings provide an important behavioral piece of evidence for the gender congruency effect in Romance languages.

## Figures and Tables

**Table 1:** Sample of stimuli in the experimental session for the PWI task.

Target Picture PIG _[CERDO]_	Condition			
	G+S+	G+S−	G−S+	G−S−
Grammatical gender el (m.)				
	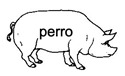	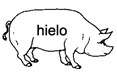	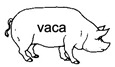	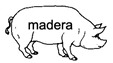
Distractors Grammatical gender of distractors	dog perro el (m.)	ice hielo el (m.)	cow vaca la (f.)	wood madera la (f.)

**Table 2:** Mean picture naming latencies by condition.

Condition	Naming Latencies (ms)	
	Mean	SD
Gender-congruent/Semantically Related (G+S+)	714	194
Gender-congruent/Semantically Unrelated (G+S−)	673	149
Gender-incongruent/Semantically Related (G−S+)	725	184
Gender-incongruent/Semantically Unrelated (G−S−)	691	165

**Table 3:** General mixed effects model of best fit for RTs with gender congruency and semantic relatedness as two predictors including estimates, confidence intervals and *p*-values. The result demonstrated that both gender congruency and semantic relatedness had a significant impact on naming latencies. “***”: *p* < 0.001 and “**”: *p* < 0.01.

Formula: naming latency ~ gender congruency (congruent vs. incongruent) + semantic relatedness (related vs. unrelated) + (semantic relatedness | participant) + (1 | item)				
**Predictors**	**RTs**			
	*Estimate*	*95% CI*	*Statistic*	*Pr(>|z|)*
(Intercept)	714.882	683.193–746.570	44.239	<0.001 ***
Gender [Congruent]	−13.526	−22.634–−4.419	−2.912	0.004 **
Semantic [Related]	38.409	17.320 – 59.498	3.571	<0.001 ***
**Random Effects**				
σ^2^	0.04			
τ_00 Participant_	2460.61			
τ_00 Item_	578.38			
τ_11 participant. SemanticRelated_	872.19			
ρ_01 participant_	−0.20			
ICC	1.00			
N _participant_	30			
N _Item_	20			
Observations	2288			
Marginal R^2^/Conditional R^2^	0.115/1.000			

## Data Availability

The data supporting the findings of this study are openly available in Open Science Framework at https://osf.io/9vjdp/?view_only=cd0afe62286b4a4f9a648ff6b307bb32 (accessed on 5 April 2023) (view-only link).

## Appendix A

**Table A1:** Table of stimuli used in picture naming task.

Target Picture	Gender	Distractor Type		Title 3	
		G+S+	G+S−	G−S+	G−S−
gato (cat)	Masculine	conejo (rabbit)	sol (sun)	rata (rat)	miel (honey)
caballo (horse)	Masculine	burro (donkey)	sello (stamp)	mula (mule)	flor (flower)
cuervo (crow)	Masculine	papagayo (parrot)	monte (mountain)	paloma (dove)	toalla (towel)
guitarra (guitar)	Feminine	trompeta (trumpet)	isla (island)	violin (violin)	zapato (shoe)
mesa (table)	Feminine	silla (chair)	araña (spider)	armario (closet)	gorro (hat)
estantería (shelving)	Feminine	mecedora (rocking chair)	bandera (flag)	sofá (sofa)	cabello (hair)
cama (bed)	Feminine	alacena (cupboard)	garra (claw)	sillón (armchair)	libro (book)
cerdo (pig)	Masculine	perro (dog)	hielo (ice)	vaca (cow)	madera (wood)
tigre (tiger)	Masculine	león (lion)	avión (airplane)	serpiente (snake)	oreja (ear)
brazo (arm)	Masculine	pie (foot)	pan (bread)	pierna (leg)	arena (sand)
boca (mouth)	Feminine	nariz (nose)	abeja (bee)	ojo (eye)	árbol (tree)
tenedor (fork)	Masculine	cuchillo (knife)	bolso (handbag)	cuchara (spoon)	pintura (painting)
tomate (tomato)	Masculine	chile (chili)	diente (tooth)	patata (potato)	piedra (stone)
abrigo (coat)	Masculine	jersey (sweater)	huevo (egg)	camiseta (shirt)	estrella (star)
manzana (apple)	Feminine	pera (pear)	bota (boot)	plátano (banana)	pescado (fish)
autobús (bus)	Masculine	tren (train)	mono (monkey)	bicicleta (bike)	llave (key)
dedo (finger)	Masculine	codo (elbow)	ratón (mouse)	palma (palm)	nave (ship)
zorro (fox)	Masculine	lobo (wolf)	papel (paper)	hiena (hyena)	lengua (tongue)
flecha (arrow)	Feminine	pistola (gun)	roca (rock)	puñal (dagger)	oso (bear)
maíz (corn)	Masculine	trigo (wheat)	gallo (rooster)	soja (soy)	carta (letter)
